# Utility of five commonly used immunohistochemical markers TTF-1, Napsin A, CK7, CK5/6 and P63 in primary and metastatic adenocarcinoma and squamous cell carcinoma of the lung: a retrospective study of 246 fine needle aspiration cases

**DOI:** 10.1186/s40169-015-0057-2

**Published:** 2015-04-21

**Authors:** Grzegorz T Gurda, Lei Zhang, Yuting Wang, Li Chen, Susan Geddes, William C Cho, Frederic Askin, Edward Gabrielson, Qing Kay Li

**Affiliations:** The Department of Pathology, The Johns Hopkins University School of Medicine, Baltimore, MD 21287 USA; The Department of Pathology and Division of Cytopathology, University of Chicago Hospitals, Chicago, IL 60637 USA; The Department of Chemistry, Magdalen College,, University of Oxford, Oxford, OX1 4 AU UK; The Department of Clinical Oncology, Queen Elizabeth Hospital, Hong Kong, SAR China; Department of Pathology, The Johns Hopkins Medical Institute, Johns Hopkins Bayview Medical Center, Baltimore, MD 21224 USA

**Keywords:** Non-small cell lung carcinoma (NSCLC), Fine needle aspiration (FNA) cytology, Cytopathology, Immunohistochemical (IHC) marker, TTF-1, Napsin A, CK7, P63 and CK5/6

## Abstract

**Background:**

Fine needle aspiration (FNA) biopsy plays a critical role in the diagnosis and staging of lung primary and metastatic lung carcinoma. Accurate subclassification of adenocarcinoma (ADC) and/or squamous cell carcinoma (SqCC) is crucial for the targeted therapy. However, the distinction between ADC and SqCC may be difficult in small FNA specimens. Here, we have retrospectively evaluated the utility of TTF-1, Napsin A, CK7, P63 and CK5/6 immunohistochemical (IHC) markers in the distinguishing and subclassification of ADC and SqCC.

**Methods:**

A total of 246 FNA cases were identified by a computer search over a two-year period, including 102 primary NSCLC and 144 primary NSCLC which had metastasized to other sites. The immunostaining patterns of TTF-1, Napsin A, CK7, P63 and CK5/6 were correlated with the histological diagnosis of the tumor.

**Results:**

In 72 primary ADCs, TTF-1, Napsin A and CK7 showed a sensitivity and specificity of 84.5%/96.4%, 92.0%/100%, and 93.8%/50.0%. In 30 primary SqCCs, CK5/6 and P63 showed a sensitivity and specificity of 100%/77.8% and 91.7%/78.3%. In 131 metastatic ADCs, Napsin A showed the highest specificity (100%), versus TTF-1 (87.5%) and CK7 (25%) but decreased sensitivity (67.8% versus 86.9% and 100%); whereas in 13 metastatic SqCCs, CK5/6 and P63 showed a sensitivity/specificity of 100%/84.6% and 100%/68.4%. Bootstrap analysis showed that the combination of TTF-1/CK7, TTF-1/Napsin A and TTF-1/CK7/Napsin A had a sensitivity/specificity of 0.960/0.732, 0.858/0.934, 0.972/0.733 for primary lung ADCs and 0.992/0.642, 0.878/0.881, 0.993/0.618 for metastatic lung ADCs.

**Conclusions:**

Our study demonstrated that IHC markers had variable sensitivity and specificity in the subclassification of primary and metastatic ADC and SqCC. Based on morphological findings, an algorithm with the combination use of markers aided in the subclassification of NSCLCs in difficult cases.

## Background

Lung cancer is the leading cause of cancer-related mortality, accounting for over 150,000 deaths per year in the United States and over 1.3 million death worldwide [1,2]. Primary lung carcinomas have been classified into small cell lung carcinoma (SCLC) and non-small cell lung carcinoma (NSCLC). The later include adenocarcinoma (50-70%), squamous cell carcinoma (20-30%) and other subtypes (<10%) [3,4]. Molecular studies of lung cancers have led to the development of personalized/targeted therapy [5-12]. An important example is the discovery of epidermal growth factor receptor gene (*EGFR*) alterations, and the successful administration of EGFR tyrosine kinase inhibitors (TKIs) in lung cancer patients whose tumor harbors *EGFR* alterations [5,10,13]. Another therapeutic target, the echinoderm microtubule-associated protein like 4(EML4)-anaplastic lymphoma kinase (ALK) fusion protein, has also been uniquely detected in a subset of adenocarcinomas [8]. Recently, more targeted therapies aimed at specific pathways and/or cell types have been developed and are in clinical trials [5,7,11]. Taken together, subclassification of NSCLC plays a critical role in the clinical management of NSCLC patients [14].

The majority of NSCLC patients present with advanced and/or metastatic disease [2,3,8]. Fine needle aspiration (FNA) cytology performed either by transthoracic and/or transbronchial procedures are important approaches to obtain tumor tissue for histological diagnosis and molecular characterization of tumors [15,16]. However FNA specimens are usually small and contain with a limited amount of tumor. Pathologists, therefore, have been faced with the challenge of an increased volume of specimens along with a concurrent demand for precise subclassification of lung cancers. For FNA specimens, the distinction between adenocarcinoma (ADC) and squamous cell carcinoma (SqCC) can be challenging due to scant tumor tissue [16-19] and several other factors, such as an obscuring tumor diathesis, crushing and drying preparation artifacts [19-21]. As a result, immunohistochemistry (IHC) has been increasingly used to aid in the subclassification of NSCLC [19-23].

Numerous recent studies have been published to address the utility of IHC markers in the diagnosis and subclassification of NSCLC using surgically resected tumor tissue [20,21,24] as well as FNA specimens [19-23,25]. However, the clinical question of how to construct an IHC panel with limited number of IHC markers and particularly how to apply commonly used IHC markers in FNA cases is still under debate. Furthermore, a daily challenge in clinical practice involves how to best use a minimal amount of tumor tissue while making an accurate and rapid diagnosis.

In this study, we have retrospectively studied five most commonly used IHC markers, TTF-1, Napsin A, CK7, P63 and CK5/6 in the subclassification of NSCLC using cytological FNA cases. We have included both primary NSCLCs and tumors of primary NSCLC which had metastasized to other body sites, and compared the sensitivity and specificity of these markers individually and in combinations. The purpose of our study is to evaluate our institutional experience and to provide an evidence-based approach for the utilization of IHC markers in daily practice.

## Methods

### Case collection

The cytological archive of the Department of Pathology at the Johns Hopkins Hospitals was searched using Boolean terms “NSCLC or ADC or SqCC” and “IHC markers”, including TTF-1, Napsin A, CK7, P63 and CK5/6 based on a period of 24 months (from 2010 to 2011). The search yielded 246 FNA cases, including 102 cases of primary lung ADC and SqCC, and 144 metastatic cases of primary lung ADC and SqCC to other sites. The available slides and the clinical information were reviewed and correlated. The study was approved by the Johns Hopkins Institutional Review Board (IRB). All study cases were annotated with available clinical information in a manner that protected patient privacy.

### Immunohistochemistry (IHC)

All of the IHC stains were performed at our clinical immunohistochemistry laboratory as previously described [22]. Briefly, the specimens were sectioned at 5 *μ*m, deparaffinized and incubated with primary antibodies. Information of primary antibodies were summarized in the Table 1. Napsin A was stained using the Leica Bondmax autostainer (Leica, Buffalo, IL). TTF-1, CK7, CK5/6 and P63 were stained using the Ventana XT autostainer. Staining characteristics were reviewed and considered along with the intensity and distribution of staining patterns. A case was considered to be positive if greater than 5% of tumor cells with an appropriate staining pattern were identified; otherwise the case was considered to be negative. In terms of specific staining patterns, coarse granular cytoplasmic staining was considered positive for Napsin A. Nuclear staining was considered positive for TTF-1 and P63. Cytoplasmic staining was considered positive for CK7 and CK5/6. Appropriate positive and negative controls were included in each assay. Care was taken not to interpret entrapped normal lung bronchial epithelium or pulmonary macrophages as a positive staining.

**Table 1:** **Summary of primary antibody information**

**Antibody**	**Source**	**Clonality**	**Clone**	**Species**	**Dilution**	**Pretreatment**
TTF-1	Ventana	Monoclonal	8G7G3/1	Mouse	Prediluted	CC1
Napsin A	Novocastra	Monoclonal	IP64	Mouse	1:800	Bond enzyme
CK7	Dako	Monoclonal	Ov-tl	Mouse	1:500	None
P63	BioCare	Monoclonal	4a4	Mouse	Prediluted	CC1
CK5/6	Ventana	Monoclonal	D5/16 B4	Mouse	Prediluted	CC1

CC1: cell conditioning 1.

### Statistical analysis

The characteristics of IHC markers were compared between different groups, including primary lung carcinomas and lung primary carcinomas metastatic to other sites. Any missing values due to loss of tumor tissue on cell blocks or if IHC was not performed were eliminated from the statistical analysis. Both individual and combinations of IHC stains were assessed to differentiate between primary and metastatic tumors. Statistical analysis was performed using GraphPad Prism software (GraphPad software Inc, La Jolla, CA). The bootstrap analysis (with 1000X re-sampling of the dataset) was also used to analyze the sensitivity and specificity for the combination of individual markers [26]. A *P* value equal and/or less than 0.05 (P ≤ 0.05) was considered as statistically significant. The sensitivity, specificity, positive predictive value (PPV) and negative predictive value (NPV) were calculated based on the final cytological diagnosis.

## Results and discussion

### Clinical information

Recent advances in the targeted therapy of lung cancers require an accurate subclassification of NSCLC. FNA-based cytological diagnosis is often the crucial first step in the diagnosis of a lung mass [7,16,18]. FNA specimens have unique problems, including small amount of tumor cells within the specimen, and potential specimen preparation artifacts. These features could affect the diagnosis and subclassification of NSCLC, so it may be difficult to subclassify the tumor by using routine hematoxylin and eosin (H & E) stained sections. Therefore, IHC markers are frequently used to aid in the diagnosis. Recent publications have already addressed the issue in the subclassification of NSCLC via small tissue biopsies and the utility of IHC markers in the surgical pathology setting [24,27-29]. However, evidence-based studies are still necessary for the determination of the optimal utilization of IHC markers, particularly in FNA specimens. In this study, we have retrospectively studied five most commonly used IHC markers, including TTF-1, Napsin A, CK7, P63 and CK5/6 in the subclassification of NSCLC in a large cohort (n = 246 cases) of cytological FNA cases.

Of 246 FNA cases, 102 cases were primary NSCLC, including 72 ADCs and 30 SqCCs, and 144 cases were primary NSCLC metastases to other body sites. Of metastatic NSCLCs, there were 131 ADCs and 13 SqCCs. The patient clinical characteristics were summarized in the Table 2. There were no gender and age differences between the primary and metastatic NSCLC (P = 0.3638 and P = 0.4110, respectively). All cases of primary NSCLCs had a clinical presentation of lung masses and radiographic diagnosis/suspicion of lung carcinomas, and 38% of the cases (39/102) had subsequent surgical biopsy and/or resection. In metastatic cases, 47.2% of cases (68/144) had a history of NSCLCs. The location of metastatic tumors is summarized in the Table 3. The most frequent metastatic body sites were local lymph nodes (45.14%, 65/144 cases) and pleural cavity (29.86%, 43/144 cases), respectively.

**Table 2:** **Clinical characteristics of patients**

**Characteristics**	**Primary lung tumor**	**Metastatic lung tumor**	**P Value**
Sex* (cases (%))			0.3638
Male	46 (45.5%)	75 (52.1%)	
Female	55 (54.5%)	69 (47.9%)	
Age (years)			0.4110
Average ± SD	65.0 ± 11.2	63.8 ± 11.3	
Median	67.0	64.0	
Range	29-85	32-89	
Adenocarcinomas	72 (70.6%)	131 (91.0%)	N/A
Squamous cell carcinomas	30 (29.4%)	13 (9.0%)	
Total cases	102	144	N/A

FNA: find needle aspiration. *For one case of a primary lung tumor, the patient sex was unknown.

**Table 3:** **Locations of metastatic tumors of the lung**

**Sites of Metastatic NSCLC**	**Cases (Percentage)**
**Adenocarcinoma (n = 131)**	
Lymph node	59 (45.0%)
Pleural fluid	40 (30.5%)
Soft tissue	8 (6.1%)
Bone	7 (5.3%)
Liver	6 (4.6%)
Pericardial fluid	4 (3.1%)
Peritoneal fluid	3 (2.3%)
Other organs	4 (3.1%)
**Squamous cell carcinoma (n = 13)**	
Lymph node	6 (46.2%)
Pleural fluid	3 (23.1%)
Soft tissues	3 (23.1%)
Other organs	1 (7.7%)

From our experience, most small biopsies lung cancer cases can be classified based on morphological evaluation, while about 30% to 40% of cases need IHC study in order to accurately classify the tumor, particularly in metastatic carcinomas. The clinical separation of the two major subtypes (ADC vs SqCC) has significant impact on the targeted therapy. Thus, in this study we have focused on the evaluation of the clinical utility of five most commonly used IHC markers in the subclassification of ADC and SqCC.

### Individual staining patterns of CK5/6 and P63 in SqCCs

**Figure 1:**
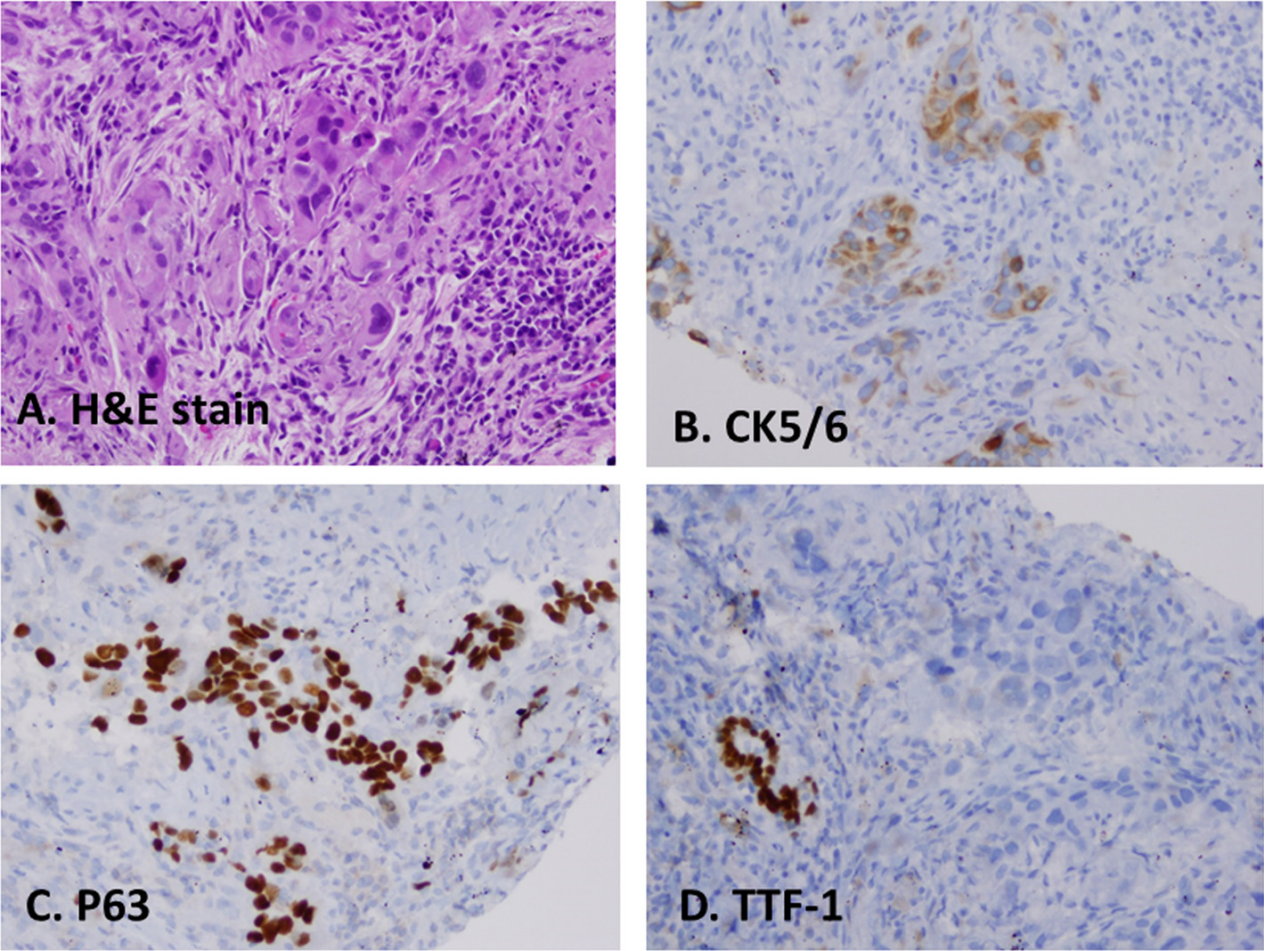
Immunostaining pattern of CK5/6 and P63 in squamous cell carcinomas. **A**, histomorphology of SqCC on H&E slide; **B**, immunostain of CK5/6 in tumor cells, **C**, immunostain of P63 in tumor cells, and **D**, stain of TTF-1 in tumor cells and entrapped normal lung bronchial epithelium. Tumor cells of SqCC are positive for CK5/6 and P63, but negative for TTF1.

The cytological features of SqCCs include pleomorphic large tumor cells with hyperchromatic nuclei, opaque or “hard” cytoplasm, intracytoplasmic processes, or other features characteristic of squamous differentiation (Figure 1). Immunostaining patterns of P63 and CK5/6 in SqCCs are shown in Figure 1 and Table 4. CK5/6 showed 100% positivity in both primary and metastatic tumors, whereas, P63 showed a 91.7% and 100% positivity in primary and metastatic tumors, respectively (Table 4). The sensitivity, specificity, positive predictive value (PPV) and negative predictive value (NPV) of CK5/6 and P63 in primary and metastatic SqCC are summarized in Table 5. In primary SqCCs, CK5/6 showed a slightly higher sensitivity than P63 (100% vs 91.7%), and similar specificity as P63 (77.8% vs 78.3%). In metastatic SqCCs, both CK5/6 and P63 revealed to have the same sensitivity (100% vs 100%), but CK5/6 showed a higher specificity than that of P63 (84.6% vs 68.4%, P < 0.05).

**Table 4:** **Immunostaining patterns in primary and metastatic NSCLC**

**NSCLC**	**P63**	**CK5/6**	**Napsin A**	**TTF-1**	**CK7**
	**Positive/total**	**Positive/total**	**Positive/total**	**Positive/total**	**Positive/total**
	**(%)**	**(%)**	**(%)**	**(%)**	**(%)**
**Primary lung carcinomas (n = 102)**					
**ADC (n = 72)**	5/23	2/9	23/25	60/71	45/48
	(21.7%)	(22.2%)	(92.0%)	(84.5%)	(93.8%)
**SqCC (n = 30)**	22/24	18/18	0/8	1/28	7/14
	(91.7%)	(100.0%)	(0.0%)	(3.6%)	(50.0%)
**Metastatic tumor of the lung (n = 143)**					
**ADC (n = 131)**	6/19	2/13	40/59	113/130	101/101
	(31.6%)	(15.4%)	(67.8%)	(86.9%)	(100.0%)
**SqCC (n = 13)**	11/11	6/6	0/3	1/8	3/4
	(100.0%)	(100.0%)	(0.0%)	(12.5%)	(75.0%)

NSCLC: non-small cell lung carcinoma. ADC: adenocarcinoma. SqCC: squamous cell carcinoma.

**Table 5:** **Performance of individual marker in primary and metastatic lung squamous cell carcinomas**

**Type**	**Primary SqCC (n = 30) vs**				**Metastatic SqCC (n = 13) vs**			
	**Primary ADC (n = 72)**				**Metastatic ADC (n = 131)**			
	**Sensitivity**	**Specificity**	**PPV**	**NPV**	**Sensitivity**	**Specificity**	**PPV**	**NPV**
**P63**	91.7%	78.3%	81.5%	90.0%	100.0%	68.4%	64.7%	100.0%
**CK5/6**	100.0%	77.8%	90.0%	100.0%	100.0%	84.6%	75.0%	100.0%

ADC: adenocarcinoma. SqCC: squamous cell carcinoma. PPV: positive predictive value. NPV: negative predictive value.

In SqCCs, we also found that TTF-1 and Napsin A could stain entrapped bronchial epithelial cells (Figure 1D) and alveolar macrophages (data not shown) rather than tumor cells. In this circumstance, tumor cells were considered negative for TTF-1 and Napsin A.

In SqCCs, P63 and CK5/6 are commonly used markers. Human *TP63* gene is located on the chromosome 3q27-29; and the expression of the gene produces the full-length protein P63 and the truncated protein P40 [22,24]. P63 can be detected in benign bronchial stem cells and in neoplastic cells with evidence of squamous differentiation [30]. CK5/6 is a high molecular weight cytokeratin and expressed in neoplasms of epithelial origin, including SqCC, mesothelial carcinoma and urothelial carcinoma [31]. P63 and CK5/6 have been used in the diagnosis of lung SqCC [23,24,27,32], however typically in much smaller cohorts (≤50 cases) [23,32] than our study here. Our data demonstrates that P63 and CK5/6 have the sensitivity and specificity of 91.7% and 78.3%, and 100% and 77.8%, respectively. We found that CK5/6 to be more sensitive in primary SqCC, and more specific for metastatic SqCC (Tables 3 and 4). Both of these markers however can be detected in subset of ADCs. For example, P63 expression can be seen in 21.7% of primary ADCs and 31.6% of metastatic ADCs (Table 4). However it usually shows weak and focal expression in contrast to strong and diffuse staining seen in SqCC. These observations arise the potential possibility of glandular differentiation in SqCC. It has also been reported that in cases of poorly differentiated ADCs, which show a decreased expression of Napsin A and TTF-1 [19,21], and a proportion of these tumors have been shown to be P63-positive [33,34]. Similarly, CK5/6 has also been detected in a subset of primary ADCs (22.2%) and metastatic ADCs (15.4%). In addition, CK5/6 has also been detected in some cases of pancreatic, endometrial and breast ADCs [31].

ΔNP63 (N-terminal-truncated protein isoform of TA63), the truncated form of P63 without the transactivation domain, can be identified by the antibody designated as P40. P40 has been reported to have better sensitivity and specificity than P63 in the identification of SqCCs. Our previous study, however, showed that in SqCC P40, P63 and CK5/6 had a sensitivity of 80.5%, 90.0% and 93.5% and a specificity of 80.0%, 89.6% and 80.0%, respectively [21].

In addition, we also found that CK7 was positive in 50% (7/14 cases) of primary SqCCs and 75% (3/4 cases) of metastatic SqCCs. However, our study only had few cases of SqCC. A further study with larger numbers is necessary to draw a conclusion.

### Staining patterns of TTF-1, Napsin A and CK7 in ADCs

**Figure 2:**
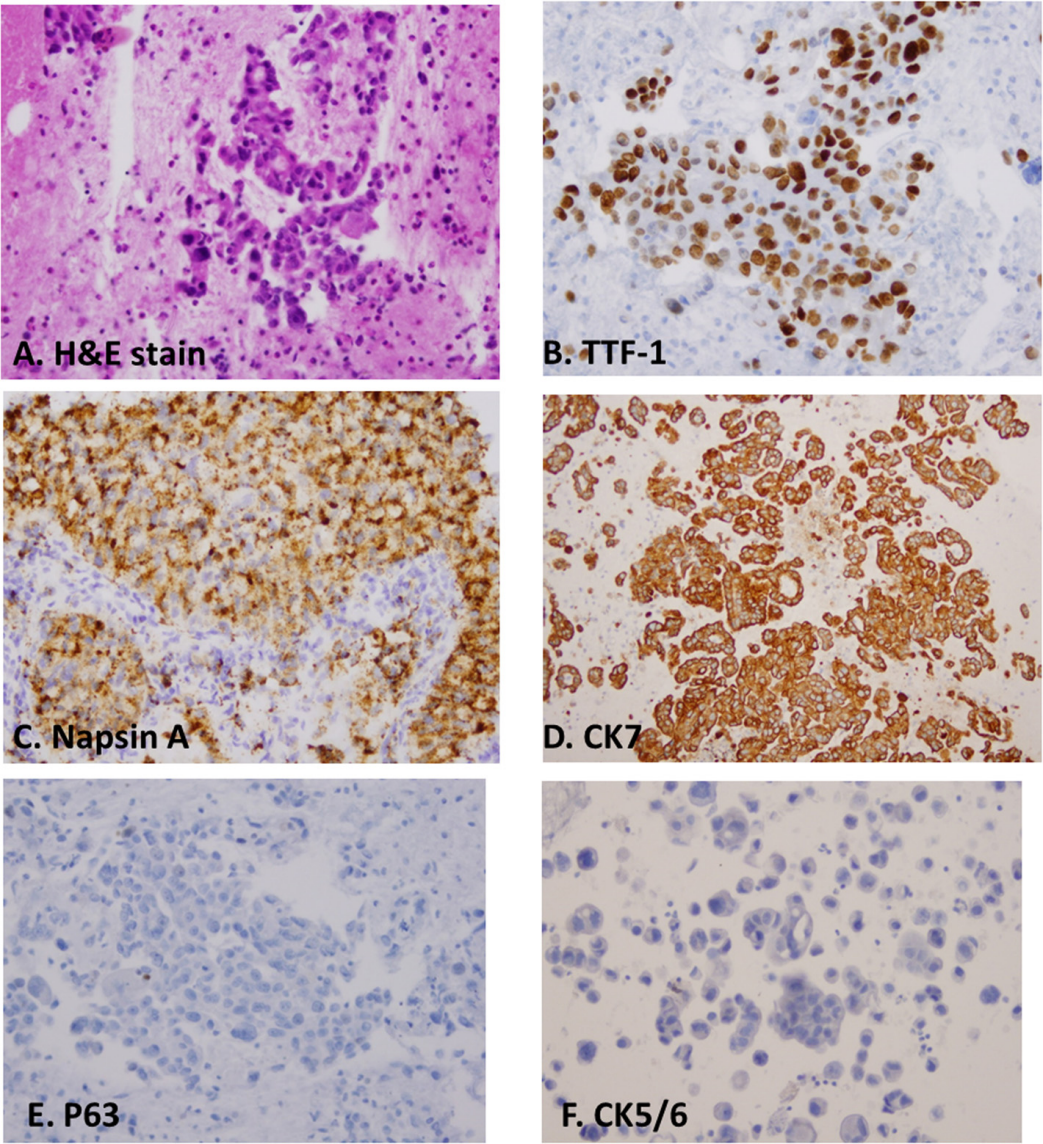
Immunostaining pattern of TTF-1, Napsin A and CK7 in adenocarcinomas. **A**, histomorphology of ADC on H&E slide; **B**, immunostain of TTF-1 in tumor cells, **C**, immunostain of Napsin A in tumor cells, **D**, stain of CK7 in tumor cells, **E**, stain of P63 in tumor cells, and **F**, stain of CK5/6 in tumor cells. Tumor cells of ADC are positive for TTF-1, Napsin A and CK7, but negative for P63 and CK5/6.

The cytological features of ADCs include clusters of tumor cells with prominent nucleoli, predominate or overt mucin production, vacuolated cytoplasm, acinar formation and other features characteristic of glandular differentiation (Figure 2). Immunostaining patterns of TTF-1, Napsin A, and CK7 in ADCs are shown in Figure 2 and Table 4. TTF-1 showed 84.5% and 86.9% positivity in primary and metastatic ADCs, whereas, Napsin A showed 92% and 67.8% positivity in primary and metastatic ADC, and CK7 showed 93.8% and 100% positivity in primary and metastatic ADCs, respectively, (Table 4). The sensitivity, specificity, PPV and NPV of TTF-1, Napsin A and CK7 in primary and metastatic ADC are summarized in Table 6. TTF-1 showed similar sensitivity between primary and metastatic ADCs (84.5% vs 86.9%, P > 0.05), but a higher specificity in primary ADCs than metastatic ADCs (96.4% vs 87.5%, P < 0.05). Napsin A showed a higher sensitivity in primary ADC than that of metastatic ADCs (92.0% vs 67.8%, P < 0.05), and the same specificity in both primary and metastatic ADC (100% for both). The sensitivity and specificity of CK7 in primary and metastatic ADCs were 93.8% and 50%, and 100% and 25%. In addition to ADCs, we also found that CK7 showed 50% and 75% positivity in primary and metastatic SqCC, respectively. Taken together, TTF-1 had a better sensitivity, and Napsin A had a better specificity for the primary lung ADCs. Whereas, CK7 showed a suboptimal specificity for lung ADCs.

**Table 6:** **Performance of individual marker in primary and metastatic lung adenocarcinomas**

**Type**	**Primary ADC (n = 72) vs**				**Metastatic ADC (n = 131) vs**			
	**Primary SqCC(n = 30)**				**Metastatic SqCC(n = 13)**			
	**Sensitivity**	**Specificity**	**PPV**	**NPV**	**Sensitivity**	**Specificity**	**PPV**	**NPV**
**TTF-1**	84.5%	96.4%	98.4%	71.1%	86.9%	87.5%	99.1%	29.2%
**CK7**	93.8%	50.0%	86.5%	70.0%	100.0%	25.0%	97.1%	100.0%
**Napsin A**	92.0%	100.0%	100.0%	80.0%	67.8%	100.0%	100.0%%	13.6%

ADC: adenocarcinoma. SqCC: squamous cell carcinoma. PPV: positive predictive value. NPV: negative predictive value.

CK7, TTF-1 and Napsin A are the most commonly used primary lung ADC markers in daily practice. Although CK7 has been used for decades to identify lung ADCs, its suboptimal sensitivity and specificity are well-known [24,27,35]. TTF-1 is a nuclear transcript factor that is expressed in epithelial cells of the lung and thyroid. In the lung, it regulates the expression of genes involved in production of surfactant. The sensitivity and specificity of TTF-1 in the identification of lung origin vary, and range from 75% to over 95% [23,27,28,36]. However, TTF-1 is also immunoreactive in several other tumors, such as thyroid neoplasms, breast adenocarcinoma, gastrointestinal carcinomas, small cell lung carcinoma (SCLC), carcinoid and, possibly but controversially [37], primary lung squamous cell carcinoma [35,38-43]. Napsin A is a relatively new marker for primary lung ADCs [44]. It is a 35-kilodalton protein that is expressed in type II pneumocytes, alveolar macrophages, and renal tubular cells [44]. Functionally, it is an aspartic protease involved in the posttranslational modification of surfactant protein B (SP-B) in type II pneumocytes [45]. The expression of Napsin A has been shown to be transcriptionally regulated by TTF-1 [46]. Previous studies using surgical resected specimens indicated that Napsin A has a better sensitivity and specificity than TTF-1 in well to moderately differentiated lung ADCs [22,23,43]. Therefore, it has been used with TTF-1 together in the differential diagnosis of lung adenocarcinomas [23,47]. Napsin A may be particularly useful in poorly differentiated ADCs, which may lose TTF-1 expression [16,19].

In our study, CK7 has a sensitivity and specificity of 93.8% and 50.0% in primary lung ADCs, and 100% and 25.0% in metastatic lung ADCs. TTF-1 has the sensitivity and specificity of 84.5% and 96.4% in primary lung ADCs, and 86.9% and 87.5% in metastatic lung ADCs. Napsin A has sensitivity and specificity of 92.0% and 100% in the primary lung ADCs, and 67.8% and 100% in metastatic lung ADCs. Taken together, all three markers revealed similar sensitivities in primary lung ADCs; Napsin A showed the best and CK7 showed the worst specificity. In metastatic ADCs, CK7 showed better sensitivity than TTF-1 and Napsin A, but the worst specificity. In our medical institutions, Napsin A was introduced to the clinical practice in 2008. Interestingly, we found that the addition of Napsin A to routine practice coincided with an increase in the diagnosis of ADC subtypes from 14% to 36% and a concurrent decrease in NOS (otherwise not further classified) subtypes from 24% to 9% among NSCLC. This observation of improved subclassification of NSCLC with the use of Napsin A has also been reported elsewhere [22,23,36,48]. All studies, however, should also address limitations of Napsin A including: (a) Napsin A is positive in some cases of SCLC and lung carcinoid [42], (b) Napsin A is positive in some cases of renal cell carcinomas [43] and (c) Napsin A also stains pulmonary macrophages [37], which need to be distinguished morphologically from tumor cells. With these caveats in mind, we show that Napsin A exhibits strong specificity for ADCs of the lung origin.

### The combination of IHC markers in NSCLCs

We also examined the utility of these IHC markers in combinations. In SqCCs, we found that 14 cases of primary and 3 cases of metastatic tumors had both CK5/6 and P63 staining. Of SqCCs with paired IHC, we found that two P63 negative SqCCs were positive for CK5/6, and one CK5/6 negative SqCC was positive for P63. In primary ADCs, we found that 24 cases were stained with TTF-1 and Napsin A, 31 cases were stained with TTF-1 and CK7, and 14 cases were stained with TTF-1, Napsin A and CK7. In metastatic ADCs, we found that 9 cases were stained with TTF-1 and Napsin A, 52 cases were stained with TTF-1 and CK7, and 49 cases were stained with TTF-1, Napsin A and CK7. Of cases with paired IHC, six Napsin A negative cases were positive for TTF1, and two TTF-1 negative cases were positive for Napsin A.

**Figure 3:**
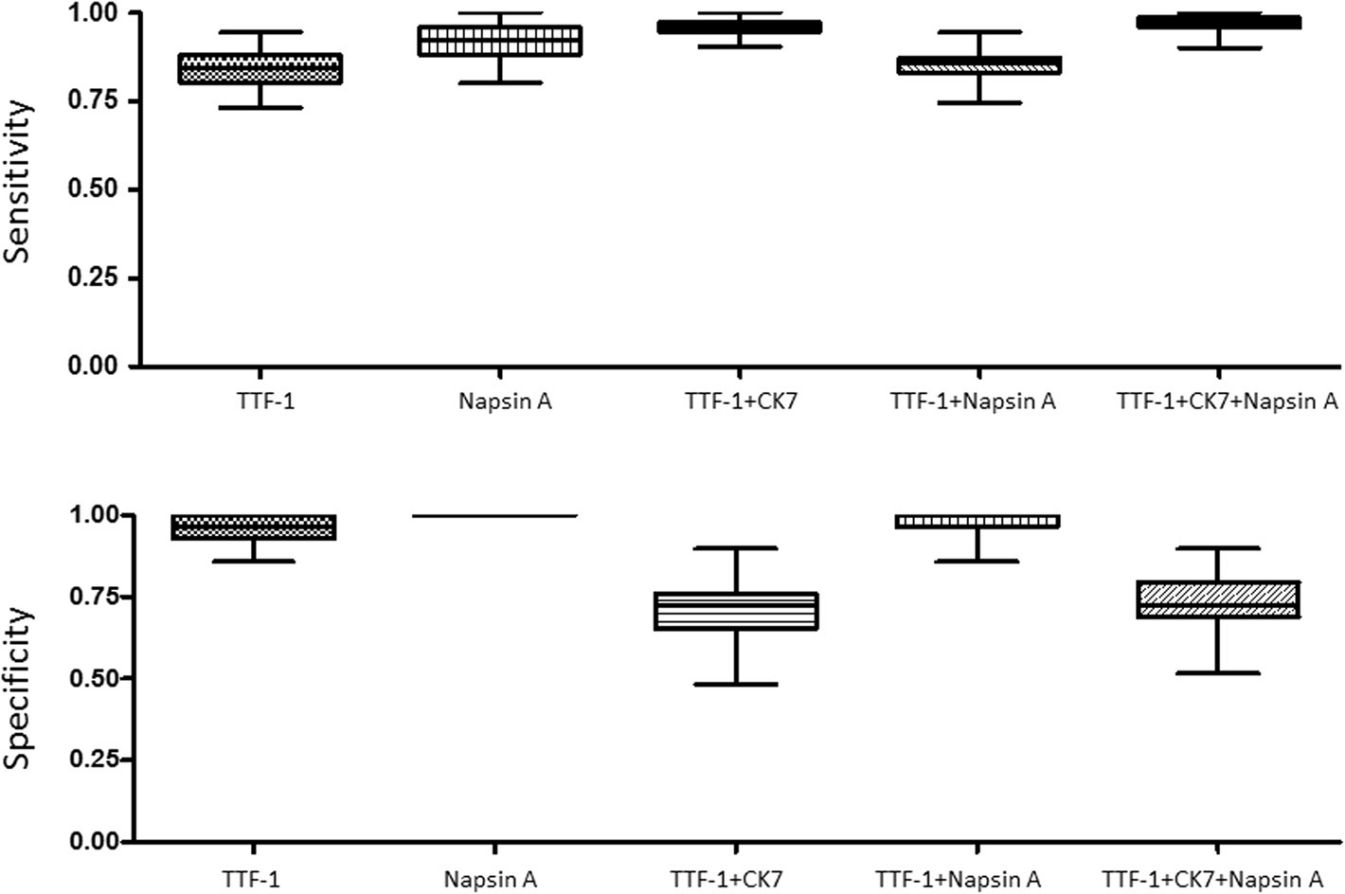
The sensitivity and specificity of IHC markers, as individual or in combination, in primary lung adenocarcinomas by the bootstrap resampling analysis.

**Figure 4:**
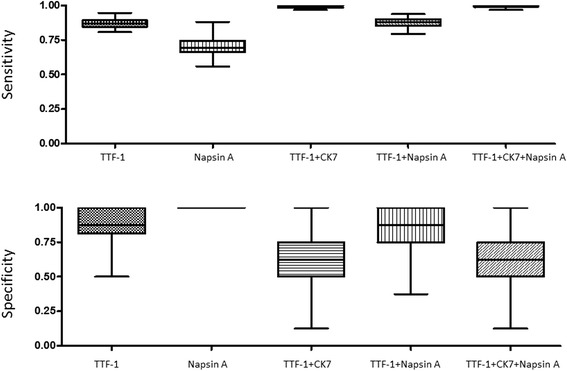
The sensitivity and specificity of IHC markers, as individual or in combination, in metastatic lung adenocarcinomas by the bootstrap resampling analysis.

To further test the sensitivity and specificity of IHC markers in combination, we used the bootstrap resampling approach to analyze both primary and metastatic NSCLCs. In ADCs, the utility of a combination of IHC markers (TTF-1, Napsin A and CK7) was compared with individual markers, with the results of primary ADCs shown in Figure 3, and metastatic ADCs shown in Figure 4. The sensitivity and specificity of combined markers were summarized in Table 7. Taken together, inclusion of TTF-1 and/or Napsin A in a panel shows an improved specificity in both primary and metastatic ADCs.

**Table 7:** **Performance of markers in primary and metastatic lung adenocarcinomas by bootstrap analysis**

**Markers**	**Sensitivity**		**Specificity**	
	**Average ± SD**	**Range**	**Average ± SD**	**Range**
**Primary ADCs**				
**TTF-1**	0.848 ± 0.0446	0.690 – 0.972	0.934 ± 0.0438	0.800 – 1.000
**Napsin A**	0.919 ± 0.0545	0.720 – 1.000	1.000 ± 0.0000	1.000 – 1.000
**CK7**	0.941 ± 0.0330	0.833 – 1.000	0.489 ± 0.1432	0.077 – 0.769
**TTF-1 + CK7**	0.960 ± 0.0231	0.718 – 0.986	0.732 ± 0.0919	0.533 – 0.967
**TTF-1 + Napsin**	0.858 ± 0.0420	0.875 – 1.000	0.934 ± 0.0417	0.833 – 1.000
**TTF-1 + CK7 + Napsin A**	0.972 ± 0.0197	0.887 – 1.000	0.737 ± 0.0850	0.500 – 0.900
**Metastatic ADCs**				
**TTF-1**	0.870 ± 0.0310	0.808 – 0.946	0.870 ± 0.1243	0.500 – 1.000
**Napsin A**	0.702 ± 0.0647	0.559 – 0.881	1.000 ± 0.0000	1.000 – 1.000
**CK7**	1.000 ± 0.0000	1.000 – 1.000	0.285 ± 0.2358	0.000 – 1.000
**TTF-1 + CK7**	0.992 ± 0.0086	0.969 – 1.000	0.624 ± 0.1662	0.125 – 1.000
**TTF-1 + Napsin**	0.878 ± 0.0298	0.794 – 0.939	0.881 ± 0.1286	0.375 – 1.000
**TTF-1 + CK7 + Napsin A**	0.993 ± 0.0072	0.969 – 1.000	0.618 ± 0.1793	0.125 – 1.000

ADC: adenocarcinoma. SqCC: squamous cell carcinoma.

**Figure 5:**
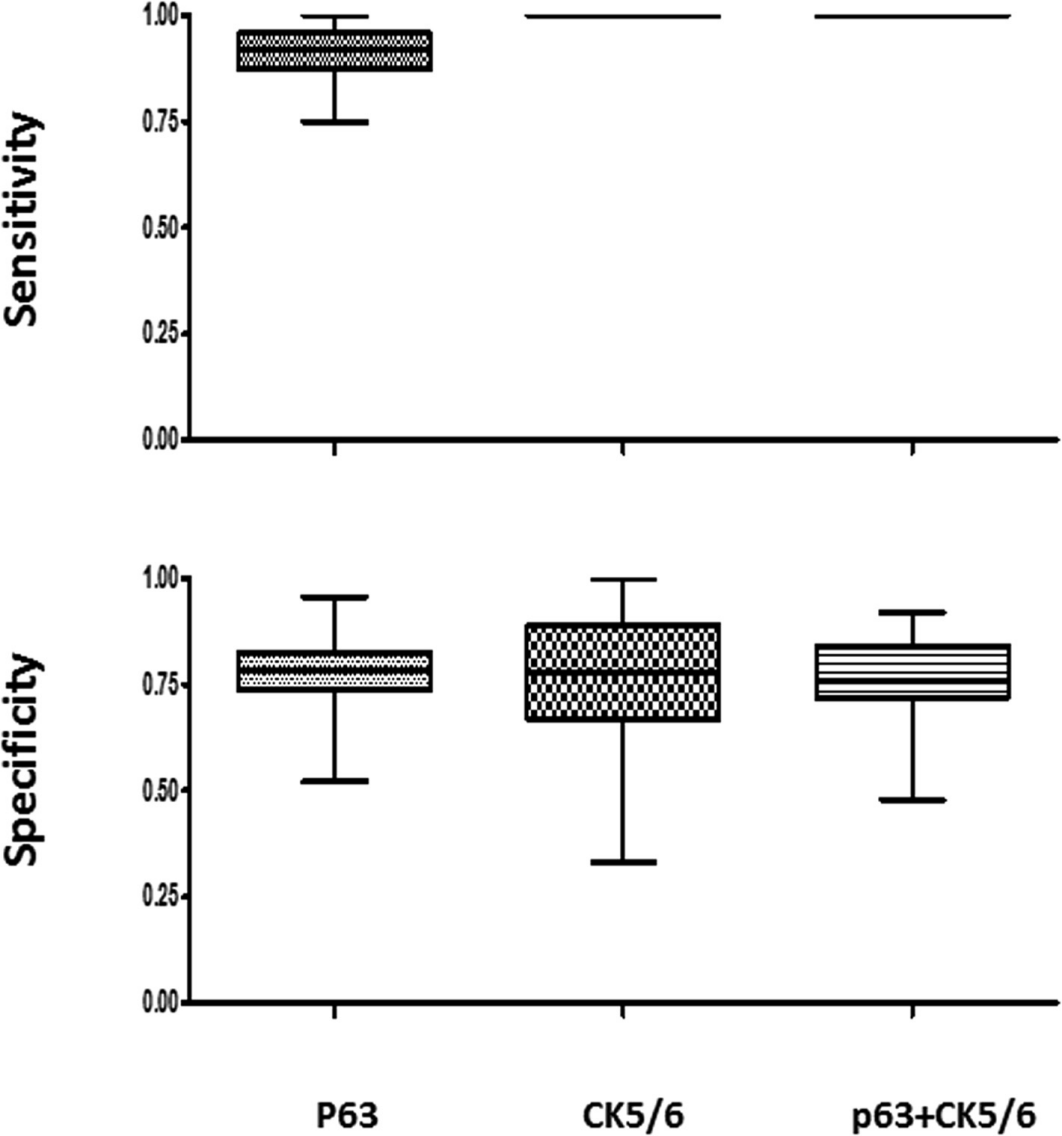
The sensitivity and specificity of IHC markers, as individual or in combination, in primary lung squamous cell carcinomas by the bootstrap resampling analysis.

**Figure 6:**
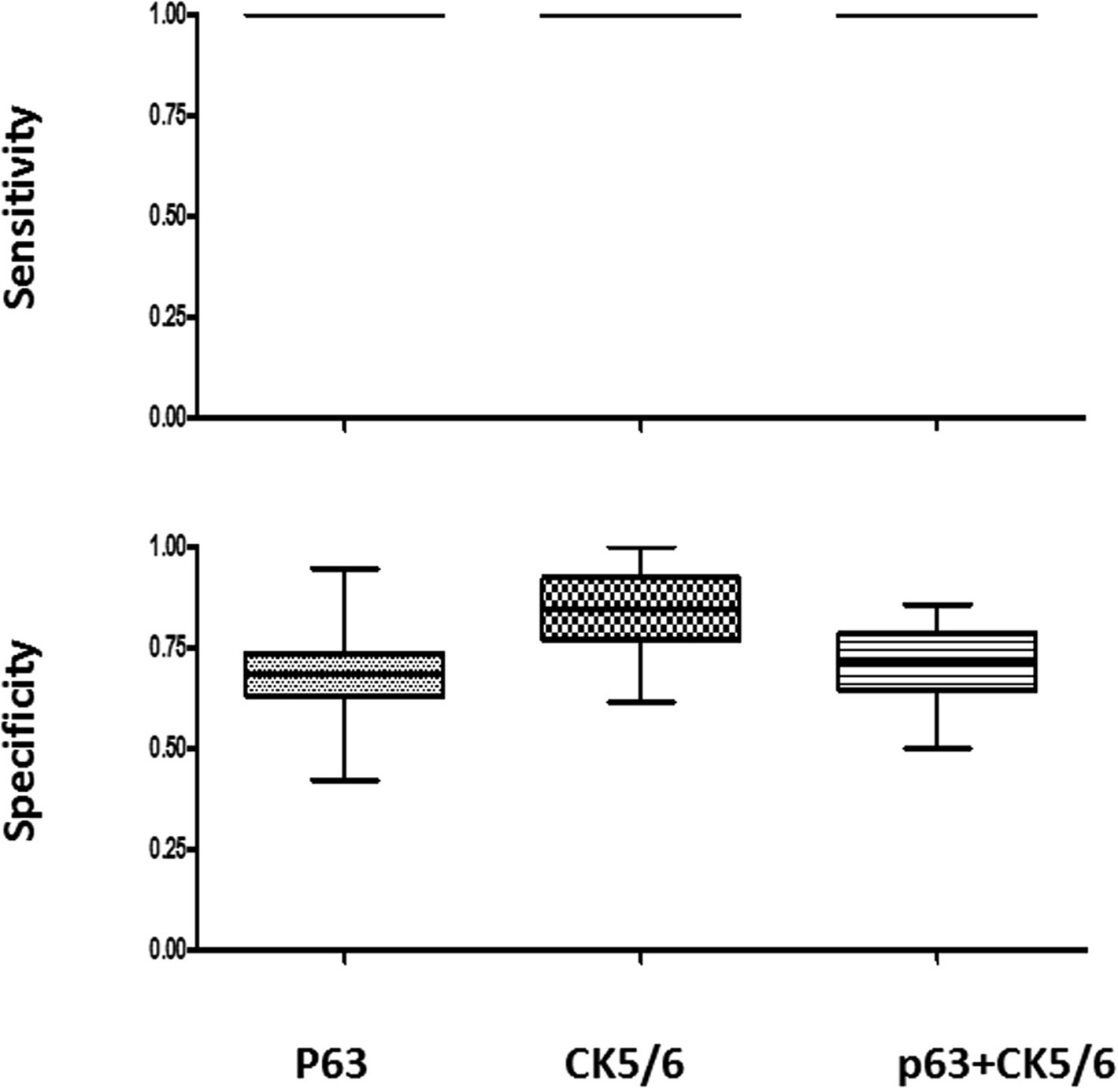
The sensitivity and specificity of IHC markers, as individual or in combination, in metastatic squamous cell carcinomas by the bootstrap resampling analysis.

In SqCCs, the utility of combinations of IHC markers P63 and CK5/6 was compared with individual markers, and results of primary SqCCs are shown in Figure 5 and metastatic SqCCs are shown in Figure 6. The sensitivity and specificity of combined markers were summarized in Table 8. Although the specificity of P63 and CK5/6 is similar in primary SqCCs, CK5/6 shows better specificity in metastatic SqCCs.

**Table 8:** **Performance of marker in primary and metastatic lung squamous cell carcinomas by bootstrap analysis**

**Markers**	**Sensitivity**		**Specificity**	
	**Average ± SD**	**Range**	**Average ± SD**	**Range**
**Primary SqCCs**				
**P63**	0.921 ± 0.0557	0.750 – 1.000	0.780 ± 0.0913	0.522 – 0.957
**CK5/6**	1.000 ± 0.0000	1.000 – 1.000	0.769 ± 0.1410	0.333 – 1.000
**P63 + CK5/6**	1.000 ± 0.0000	1.000 – 1.000	0.766 ± 0.0872	0.480 – 0.920
**Metastatic SqCCs**				
**P63**	1.000 ± 0.0000	1.000 – 1.000	0.691 ± 0.1068	0.421 – 0.947
**CK5/6**	1.000 ± 0.0000	1.000 – 1.000	0.839 ± 0.1009	0.615 – 1.000
**P63 + CK5/6**	1.000 ± 0.0000	1.000 – 1.000	0.716 ± 0.0901	0.500 – 0.857

ADC: adenocarcinoma. SqCC: squamous cell carcinoma.

The bootstrap method is a computer simulation technique based on random re-sampling of a dataset and subsequent analysis of the data distribution [26]. It is commonly used to estimate confidence intervals, the bias and variance of the actual dataset. Our data demonstrated that the combination of TTF-1 and Napsin A showed the highest specificity among three different combinations of TTF-1 + CK7, TTF1 + Napsin A and TTF-1 + CK7 + Napsin A in the identification of both primary and metastatic lung ADCs (Table 7). The specificity of P63 and CK5/6 is similar in primary SqCCs, CK5/6 shows better specificity in metastatic SqCCs (Table 8).

**Figure 7:**
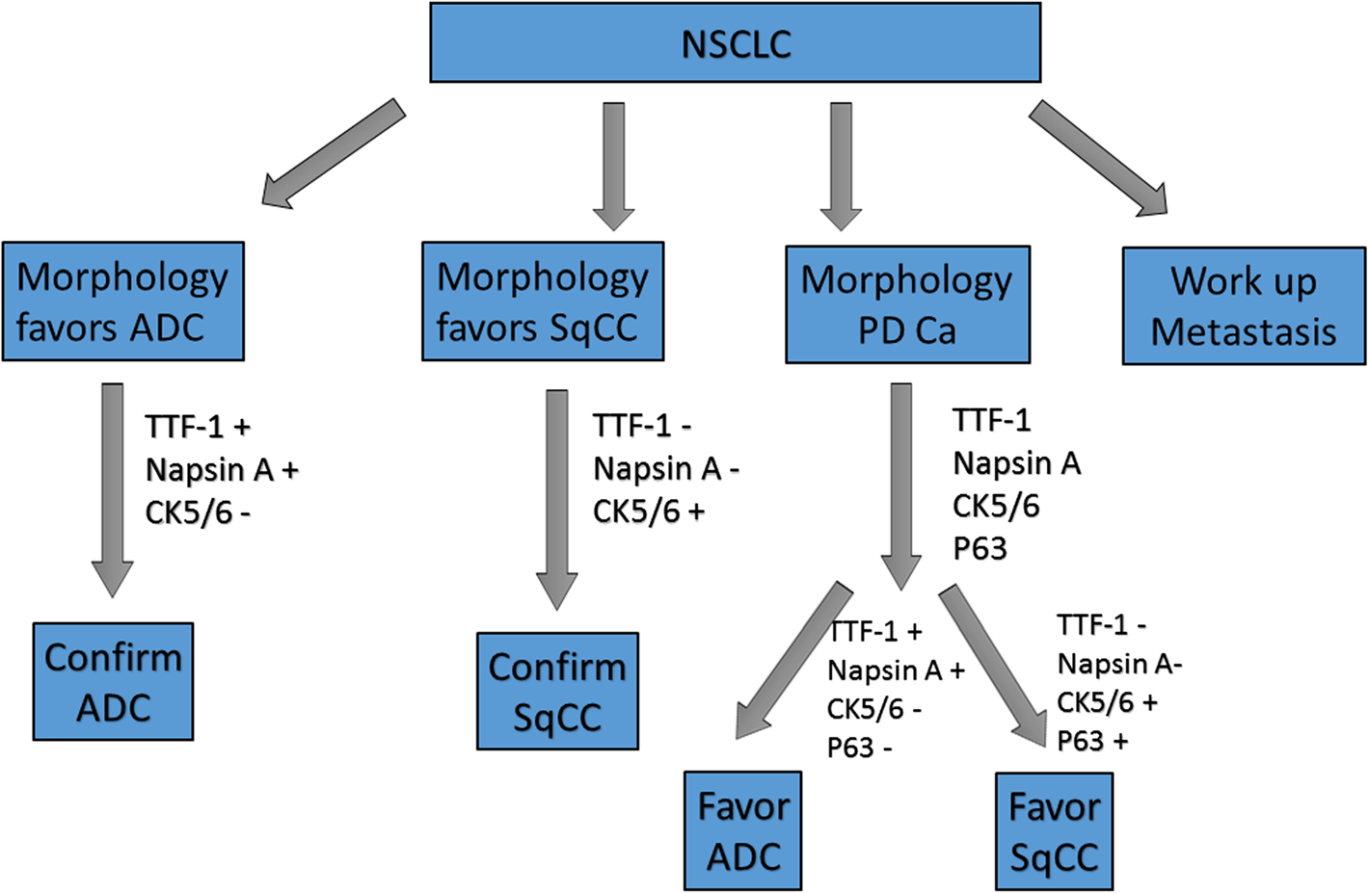
Outline of an algorithmic approach in the subclassification of NSCLC using FNA cases. In the algorithm, the evaluation of cytological morphology in the conjunction of immunostaining patterns is necessary for the final diagnosis of the tumor and/or further decision-making steps.

Based on the findings that TTF-1 and Napsin A have a high sensitivity and specificity for the diagnosis of primary lung ADCs, and CK5/6 stain is highly sensitive and specific for squamous differentiation, we outlined an algorithmic approach in the subclassification of NSCLC using FNA cases (Figure 7). In the algorithm, the evaluation of cytological morphology in the conjunction of immunostaining patterns is necessary for the final diagnosis of the tumor and/or further decision-making steps. For example: (a) ADCs should be favored for cases with both Napsin A and TTF-1 positivity; alternatively, either TTF-1 or Napsin A positivity, alongside CK5/6 negativity, (b) SqCCs should be favored for cases with CK5/6 positivity alongside Napsin A and TTF-1 negativity. The use of CK7 and P63 was not necessary to improve the identification of either ADC or SqCC. However, in poorly differentiated NSCLCs and/or adenosquamous carcinomas more IHC markers are needed. This approach may potentially improve the detection specificity, and may prove to be of diagnostic significance, as patients with poorly differentiated carcinomas may benefit from molecular screening for *EGFR*, *KRAS* or *ALK* mutations. As a note of caution, additional IHC markers are necessary to identify the origin of the tumor in cases of non-pulmonary carcinomas metastatic to the lung, and a careful clinical evaluation in these settings is also necessary [49]. The study by Su YC, et al., reported that CK7 was frequently detected in breast carcinoma in addition to lung ADCs, and CK20 was significantly more frequently detected in gastrointestinal carcinomas [50]. The other study by Kargi A, et al., reported that a subset of SCLC had a positive immunostaining pattern of TTF1 and negative immunostaining pattern of P63 [51]. Therefore, the interpretation of IHC patterns should be correlated with the morphological findings.

## Conclusions

The 2011 IASLC/ATS/ERS lung adenocarcinoma classification recommends using a single adenocarcinoma marker (TTF-1 or Napsin A) and a single squamous marker for NSCLC classification in small biopsy or cytology specimen in the absence of definitive glandular or squamous morphology to reserve tissue [3]. Our cohort showed that TTF-1 and Napsin A tend to have variable sensitivity and specificity in primary and metastatic adenocarcinoma of the lung. In metastatic adenocarcinoma, the lower sensitivity of Napsin A may lead to an inappropriate exclusion of patients from receiving the benefits of targeted therapy. Therefore combined use of TTF-1, Napsin A and CK7 could be considered in problematic cases.

In summary, the FNA-based sampling could present a unique set of diagnostic challenges, such as small amount of tumor cells, obscuring effect of tumor necrosis, the need to assess samples from different areas/multiple needle passes on a single slide and difficulty in quantifying the degree or extent of IHC staining. We evaluated the most commonly used five IHC markers, including TTF-1, Napsin A, CK7, P63 and CK5/6 in the subclassification of NSCLC. Based on our findings, we propose an algorithmic approach utilizing a panel of IHC markers for subclassification of NSCLC. Our step-wise approach allows prioritization of markers if the amount of tissue or resources is limited, in order to optimally conserve tissue for future molecular testing of the lung carcinoma. This subclassification approach has the potential benefit to improve the IHC diagnostic utilization. A further prospective study using independently collected cohort is necessary to validate our approach.

## Abbreviations

NSCLC: Non-small cell lung carcinoma
SCLC: Small cell lung carcinoma
ADC: Adenocarcinoma
SqCC: Squamous cell carcinoma
FNA: Fine needle aspiration
IHC: Immunohistochemistry
H&E: Hematoxylin and eosin
TTF: Thyroid transcription factor
CK: Cytokeratin
EGFR: Epidermal growth factor receptor
TKI: Tyrosine kinase inhibitors
ALK: Anaplastic lymphoma kinase
EML4: Echinoderm microtubule-associated protein like 4
IRB: Institutional review board
PPV: Positive predictive value
NPV: Negative predictive value
